# Analysis of proportions using arcsine transform with any experimental design

**DOI:** 10.3389/fpsyg.2022.1045436

**Published:** 2023-01-30

**Authors:** Louis Laurencelle, Denis Cousineau

**Affiliations:** ^1^Département des sciences de l'activité physique, Université du Québec à Trois-Rivières, Trois-Rivières, QC, Canada; ^2^École de psychologie, Université d'Ottawa, Ottawa, ON, Canada

**Keywords:** proportions, arcsine transform, factorial designs, repeated-measure designs, Anscombe transform

## Abstract

**Introduction:**

Exact tests on proportions exist for single-group and two-group designs, but no general test on proportions exists that is appropriate for any experimental design involving more than two groups, repeated measures, and/or factorial designs.

**Method:**

Herein, we extend the analysis of proportions using arcsine transform to any sort of design. The resulting framework, which we have called *Analysis of Proportions Using Arcsine Transform* (ANOPA), is completely analogous to the analysis of variance for means of continuous data, allowing the examination of interactions, main and simple effects, *post-hoc* tests, orthogonal contrasts, et cetera.

**Result:**

We illustrate the method with a few examples (single-factor design, two-factor design, within-subject design, and mixed design) and explore type I error rates with Monte Carlo simulations. We also examine power computation and confidence intervals for proportions.

**Discussion:**

ANOPA is a complete series of analyses for proportions, applicable to any design.

## Introduction

Proportions and their related representation, percentages, are ubiquitous. Some claim that “the percentage is the most useful statistic ever invented” (Buchanan, 1974, p. 629) but it may not be the most intuitive as we get statements such as “Baseball is 90% mental and the other half is physical!” (attributed to Yogi Berra; Knapp, 2009). Despite their pervasiveness, analyzing proportions is still difficult for most researchers, with no agreed-upon techniques, no agreed-upon confidence intervals, and no agreed-upon approach to statistical power planning.

An analysis of proportions exists for 1-group designs, performed using a binomial test or its normal approximations, and also for 2-group designs (Liddell's maximum-likelihood test, Liddell, 1976; Laurencelle's exact Bayes test, Laurencelle, 2005, 2021a). Copas (1989) examined the case with a single factor having any number of groups (commonly called a one-way design). Some advocated the use of regression with a binomial distribution, also known as logistic regression or beta distribution, underlying the data (Grizzle et al., 1969; Crowder, 1978; Allison, 1999; Beh, 2001; Agresti, 2003; Ferrari and Cribari-Neto, 2004). However, these approaches are not based on exact mathematical foundations, have limitations, and cannot be easily generalized to situations involving factorial designs, repeated measures (such as pre-post designs), and mixed designs. More critically, none of them can be additively decomposed to run contrast analyses or multiple comparison tests.

Instead of testing proportions, an alternative is to aggregate the raw data through a function, let us call it *f*, that makes the observations amenable to statistical testing. In studies where proportions are examined, the raw datum *x*_*i*_ can have two values: success or failure. The number of successes in a group of observations is noted as *s* and the number of failures is noted as *r*, for a total group size of *n* = *s* + *r*. One frequently-used aggregate, *p* = *f(s, n)* = *s / n*, is called the observed proportion, with *p* between 0 and 1. It is equivalent to a simple average, $p=\overset{n}{\underset{i=1}{\sum }}x_{i}/n$, when the scores are coded with “1s” and “0s,” as is commonly done (and likewise, with this coding, $s=\overset{n}{\underset{i=1}{\sum }}x_{i}$). The difficulties with this aggregate are 2-fold because: (i) the variance of *p* is linked to its expectation and (ii) its distribution is skewed when the expectation (or population proportion) differs from 0.50. Thus, comparing groups with different proportions necessarily entails heterogeneous variances, in violation of homoscedasticity, a condition required of most statistical procedures.

An early solution, proposed by Zubin (1935) following preliminary work on trigonometric transforms by Fisher (1922), is to use a different aggregate function *f* that stabilizes variance, making it a constant independent of the observed proportion, and that normalizes the sampling distribution, bringing it closer to a normal (Gaussian) distribution (Johnson and Kotz, 1969; Chen, 1990). Zubin's original proposal was based on the arcsine transform, $f\left(s,n\right)=\sin^{-1}\left(\sqrt{s/n}\right)$, with results—expressed in radians—ranging from 0 to π /2 ≈ 1.57. This proposal was later refined by Anscombe (1948), who proposed the aggregate function *A*, defined as


(1) $$\begin{array}{l} A=f\left(s,n\right)=\sin^{-1}\left(\sqrt{\frac{s+\frac{3}{8}}{n+\frac{3}{4}}}\right) \end{array}$$

Using this transformation, and for large *n* (we will return to this later), the asymptotic variance of *A* is 1 / (4 (*n* + ½)), independent of the population proportion. Because this is a theoretical variance, we note it as var_th_(*A*). See Freeman and Tukey (1950) and Chanter (1975) for alternatives to Equation 1; also see Wang and Fingert (2012) and Laurencelle (2021b), who reviewed the properties of these transformations, and Laurencelle (2021c), who reviewed their distributional properties.

The strongest advantage of using the arcsine transform is that its asymptotic variance (for large *n*) is known and does not have to be estimated from the data (Gabriel, 1963). As such, and because the error variance of the transform is independent of the data, a normal *z* test can be proposed for single-group and two-group designs for the null hypothesis of no difference (examined in Laurencelle, 2021a,b,c).

In what follows, we show that a wide range of tests is actually achievable, reproducing the whole array of those made possible by implementing the ANOVA logic (extending Gabriel, 1963). Building on the ANOVA logic, interactions, main effects, simple effects, *post-hoc* tests, contrast effects, et cetera, can be tested on transformed proportions. This framework is called herein ANOPA, which stands for *Analysis of Proportions Using Arcsine Transform*. It is based on formal mathematical demonstrations; the purpose of this article is to show that it constitutes a complete framework for analyzing proportions in any design.

In the following three sections, we illustrate how to perform a test when there is a single factor with *p* levels (a one-way ANOPA), when there are two crossed factors (a two-way ANOPA), and when there is a repeated-measures factor. On the OSF site accompanying this text, https://osf.io/gja9h/ (Cousineau and Laurencelle, 2022), we provide a fourth illustration with a mixed design (within-between two-way ANOPA). We then consider statistical power when planning experiments whose dependent variable is a binary outcome (e.g., success or failure). Finally, we show how to obtain confidence intervals that can serve as error bars in summary plots.

Hereafter, factors are noted with abstract upper-case letters, P and Q, and their number of levels with italic lower-case *p* and *q* (to avoid any confusion, the *p*-value will always be presented with the qualifier “-value”). For each of the following sections, the reader can find the relevant R code to perform the computations and make the plots on OSF at: https://osf.io/gja9h/. Herein, we present all the results with four decimals, even though this level of precision is unrealistic in small samples (see Altman and Bland, 1996; Cousineau, 2020), only so that interested readers can check their computations. The first three examples are based on fictitious data.

## A single factor with *p* levels

### An illustration

**Figure 1:**
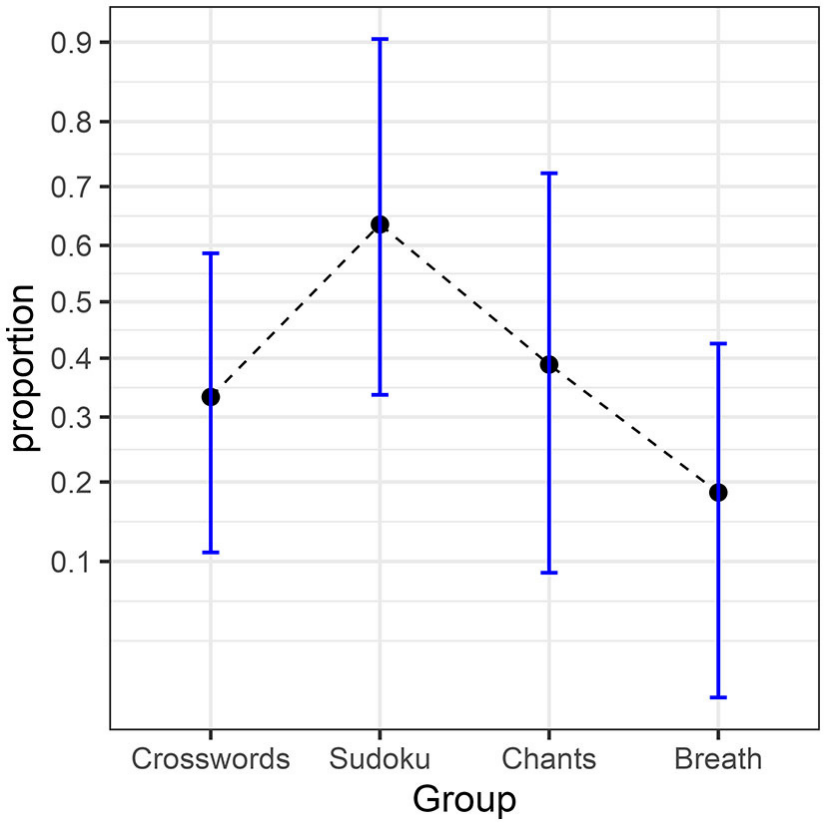
Proportions from the incubation experiment. The error bars show difference-adjusted 95% confidence intervals of the proportions (obtained with the package superb for R, Cousineau et al., 2021, as explained in the OSF website: osf.io/gja9h, folder *FirstIllustration*).

The following example on “incubation” is to be considered, where 97 participants are given a difficult problem to solve but have to complete an unrelated, distracting task for 5 min before providing a solution. The problem is to find the optimal apartment to rent from lists of attributes describing four apartments, with some attributes being desirable and others being neutral or unappealing. The dependent variable is whether the participant succeeded in determining the optimal apartment or not. In one group, participants are distracted by trying to complete a crossword puzzle; in a second group, they tackle a Sudoku; in a third, they chant a chakra and, in the last, they simply concentrate on their breathing. The results are compiled in Table 1 and illustrated in Figure 1 (how the confidence intervals were obtained will be discussed in the last section).

**Table 1:** Number of successes and group size for the four groups of the incubation experiment along with proportions (*p*) and Anscombe-transformed scores (A).

	**Number of Success (*s*)**	**Number of participants (*n*)**	***p* = *s/n***	** $A=\sin^{-1}\left(\sqrt{\frac{s+\frac{3}{8}}{n+\frac{3}{4}}}\right)$ **
Crosswords	10	30	0.3333	0.6198
Sudoku	14	22	0.6364	0.9188
Chants	7	18	0.3889	0.6779
Breath	5	27	0.1852	0.4557
Variance				0.0368

As can be seen, the two most discrepant proportions are 0.64 (Sudoku condition) and 0.19 (Breathing condition). The difference-adjusted 95% confidence interval of the former [0.34, 0.90] does not contain the later result (and vice versa, 0.64 is not contained in [0.01, 0.43]), suggesting a significant difference (see Baguley, 2012; Cousineau et al., 2021, for the purpose of difference adjustment). To formally determine if there is any significant influence of the unrelated tasks on the success rate, we ran a one-way ANOPA as follows.

This analysis is very similar to an ANOVA except that it does not bear on mean results but on Anscombe-transformed aggregates. Similarly to ANOVAs, the sum of squares of factor P and its mean square is obtained with:


(2) $$\begin{array}{l} {\text{SS}}_{\text{P}}=\overset{p}{\underset{i=1}{\sum }}{\left(A_{i}-\bar{A}\right)}^{2}=\left(p-1\right)\text{ var}\left(A_{i}\right) \end{array}$$

(3) $$\begin{array}{l} {\text{MS}}_{\text{P}}={\text{SS}}_{\text{P}}/\left(\text{p}-1\right)=\text{var}\left(A_{i}\right) \end{array}$$

in which *A*_*i*_ denotes the aggregate score of the *i*^*th*^ group, $\bar{A}$ denotes the average *A*_*i*_, var(*A*_*i*_) is the variance across the *A*_*i*_ scores, *SS*_P_ is the sum of squares, and *MS*_P_ the mean square based on *p* − 1 degrees of freedom. Note that the MS_P_ variance is not multiplied by the number of participants, contrary to standard ANOVA.

For the error term, we use the fact that the variance of Anscombe-transformed data is known theoretically, depending only on the group sizes, i.e., ${\text{var}}_{\text{th}}\left(A_{i}\right)=1/\left(4\left(n_{i}+\frac{1}{2}\right)\right)$, where *n*_*i*_ is the number of observations in the *i*th group, so that


(4) $$\begin{array}{l} {\text{MS}}_{e}=\text{mean}({\text{var}}_{\text{th}}\left(A_{i}\right))=\frac{1}{p}\overset{p}{\underset{i=1}{\sum }}\frac{1}{4\left(n_{i}+\frac{1}{2}\right)} \end{array}$$

a very close approximation is to use $1/\left(4\left(\tilde{n}+\frac{1}{2}\right)\right)$ where *ñ* is the harmonic mean of the groups' sizes. For the error variance, there is no degree of freedom because this variance is not measured from the data; it is determined mathematically by the transformation used. The only other situation where an error variance has no uncertainty is when the entirety of the population has been measured; by analogy with this situation, we will state that the degrees of freedom of the error variance are infinite. Finally, the *F* ratio is obtained as usual with


(5) $$\begin{array}{l} F_{\text{P}}=\frac{{\text{MS}}_{\text{P}}}{{\text{MS}}_{e}} \end{array}$$

We used a subscript P to indicate that this *F*-value is related to the factor P; however, in articles, *F* ratios are generally reported without a subscript.

The fact that the mean squared error decreases with an increased sample size makes ANOPA more powerful at detecting effects when samples are larger. Indeed, dividing a mean squared effect by a smaller mean squared error term yields a larger *F*, which is more likely to exceed a decision threshold.

We summarize the results of the study on incubation in Table 2. The test statistic is *F*_(3, ∞)_ = 3.51. Depending on the software or handbook used, it can be a bit difficult to get the *p*-value corresponding to an *F* with “infinite” denominator degrees of freedom (although it is sometimes given in the last line of some tables listing critical values). However, for such a quotient *F*, the multiplication by the degrees of freedom of the numerator yields a chi-square variate (Forbes et al., 2010), so the *p*-value for *g*_P_ = (*p*−1)*F*_*p*_ can be more easily obtained from a chi-square distribution with *p* – 1 degree of freedom. Either way, we find a significant *p* = 0.0145, confirming the impression we got from Figure 1.

**Table 2:** One-way ANOPA table.

	**SS**	**df**	**MS**	***F***	***p*-value**
Group (P)	0.1104	3	0.0368	3.5124	0.0145
Error	-	∞	0.0105		

Using William's correction *c*_P_ = 1.0086, we obtain *F*_(3, ∞)_ = 3.4825, *p* = 0.0151.

A further look at the data using Tukey's HSD paired comparison technique shows that the sole pairwise difference reaching significance (*q* = 4.524, *p*-value < 0.01) is between the Sudoku and Breath conditions (see the OSF page for computations using R).

### Expressing effect sizes

A convenient measure of effect size in this design is the *f*^2^, the quotient of the effect's variance per observation over the error variance. The *f*^2^ measure can be converted to a proportion of variance, an eta-square measure, with η^2^ = *f*^2^/(1 + *f*^2^).

To estimate *f*^2^ from the data,


(6) $$\begin{array}{l} {\hat{f}}^{2}=\frac{{\hat{\sigma }}_{\text{P}}^{2}}{{\hat{\sigma }}_{e}^{2}}\approx \frac{\text{M}{\text{S}}_{\text{P}}}{\tilde{n}\text{ M}{\text{S}}_{e}} \end{array}$$

can be used, where ${\hat{\sigma }}_{\text{P}}^{2}$ is obtained from the variance of the Anscombe-transformed scores as before, var(*A*_*i*_) = MS_P_, and where ${\hat{\sigma }}_{e}^{2}$ estimates the error variance, that is, ${\hat{\sigma }}_{e}^{2}=\tilde{n}\times MS_{e}$. In other words, ${\hat{f}}^{2}$ is identical to Equation (5) except that the impact of the number of participants in each cell is removed to have a standardized effect size.

In the present example, we already had ${\hat{\sigma }}_{\text{P}}^{2}$ = 0.0368; we additionally found that ${\hat{\sigma }}_{e}^{2}$ = 23.3399 × 0.0105 = 0.2446, from which we obtain ${\hat{f}}^{2}=0.1505$ and ${\hat{\eta }}^{2}=0.1308$. The magnitude of this effect size is to be appraised as usual (here, a fairly large effect).

### Type I error rates in single-factor designs

To get a general idea of the effectiveness of the one-way ANOPA to control for false rejection of the null hypothesis, we ran a simulation study in which we estimated the empirical type I error rate. We varied the total sample size $n_{Total}=\overset{p}{\underset{i=1}{\sum }}n_{i}$. For small samples, the Anscombe transformation does not fully normalize the data, and the sampling variance stays somewhat below the theoretical variance (Laurencelle, 2021b). As the results will show, one-way ANOPA is conservative when the total sample size is not large enough. We also varied the number of groups *p* from 2 to 5 together with the hypothesized population probability π of success, which was equal for all groups in each simulation run as we were examining the incorrect rejections of the null hypothesis. Finally, to explore unequal group sizes, we increased (or decreased) the probability of being in the first group (the prevalence rate, herein just called prevalence, noted ψ) ranging from 0.5 (half as small) to 2.0 (twice as large). The prevalence of the other *p* – 1 group was increased or decreased accordingly. With this approach, the total sample size is fixed, but the amount of data within groups fluctuate randomly across simulations. In total, there were 4 (number of conditions *p*) × 4 (population proportion π) × 5 (prevalence ψ) × 12 (samples sizes *n*) = 960 conditions each replicated 500,000 times. Appendix A on OSF at https://osf.io/gja9h/, folder *Appendices* provides the details of the simulations.

**Figure 2:**
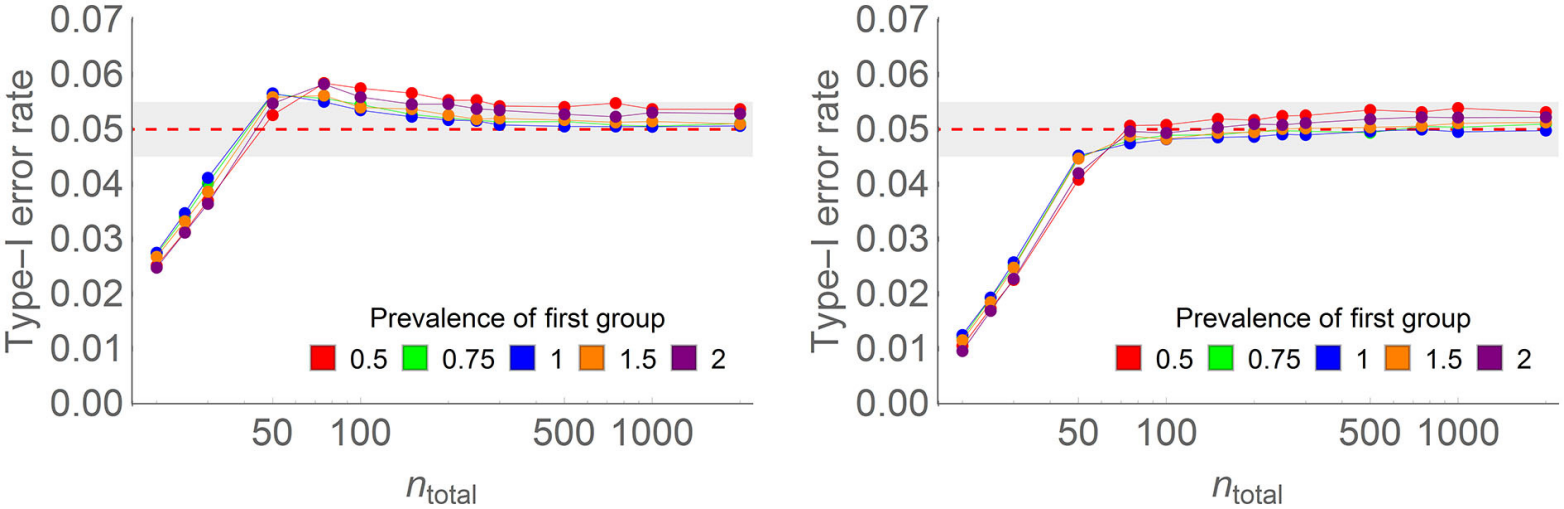
Type I error rate of the one-way ANOPA with **(on the right)** the correction factor and **(on the left)** without the correction factor in some of the conditions of the full simulations (here, the number of groups is four, the population proportion is 0.3, the prevalence of the last group relative to the other groups ranges from 0.5 to 2 (colored lines), and total sample sizes on the horizontal axis vary from 20 to 2,000 on a log scale. Each point is based on 500,000 simulations. For instance, with *n* = 100 and four groups, prevalence of 1, there were on average 25 participants per group.

Figure 2 shows some of the results (left panel) as well as results with a correction factor (right panel; the correction factor is explained in the following paragraph). The complete results with and without the correction factor are found in Appendix A.

Overall, one-way ANOPA reaches a type I error rate of 0.05 when the total sample size *n*_*Total*_ is large enough for the sampling variances to match the theoretical variances. When samples are too small, the test is conservative. See below for a rule of thumb regarding sufficient sample size.

The results also show that when *n*_*Total*_ is slightly higher than sufficient, type I error rates momentarily rise over 0.05, reaching ~0.06 rejection rate, before returning to 0.05 for a larger *n*_*Total*_. This undesirable trend is small and can be ignored. If it is important that type I error rates do not exceed the decision threshold α, a correction factor may be used. A correction factor obtained from Williams's (1976) examination of the χ^2^ distribution is given by:


(6a) c=1+p2-16 n˜(p-1)

where *ñ* is the harmonic mean of the number of participants in each group, *p* in the numerator is the number of cells in the design, and *p* − 1 in the denominator is the degree of freedom. This correction factor is to be used to divide the *F* or chi-square statistics before assessing the *p*-value. This correction factor is higher than 1 (reducing the test statistic) and tends to 1 (no reduction) for large samples. This correction factor rapidly becomes immaterial for non-trivial small groups (for example, it is worth 1.0086 for the illustration of this section).

As seen in the right panel of Figure 2, the correction factor eliminates the momentary excess in the type I error rates, with most conditions tending smoothly toward 5%. The one exception is when the population proportion is very small (0.1) or very large (0.9), as all the results are mirror images for proportions above 0.5. Prevalence had an adverse but small effect on the type I error rate: whether one group is overly represented (prevalence of 2 to 1 relative to the other groups) or underrepresented (prevalence of 0.5 to 1 relative to the other groups) in the total sample, the type I error rate increases to about 5.3%. For the other three prevalence levels (0.75, 1, and 1.5), there is no visible deviation from 0.05. Finally, the correction factor enhances the conservatism of the test for very small samples.

### Sufficient sample sizes

Following estimates of the total sample size at which corrected type I error rates reached α, we derived this rough rule of thumb from the simulations to determine what the total sample size should be:


(7) $$\begin{array}{l} n_{\text{sufficient}}=\left\lceil 20p+50\times \;|\sin^{-1}\left(\sqrt{p_{\text{extreme}}}\right)-\sin^{-1}\left(\sqrt{0.5}\right)|\right\rceil \end{array}$$

where *p*_extreme_ is the most extreme proportion observed (away from 0.5), *p* is the number of groups, and ⌈*x*⌉ denotes the first integer greater than or equal to *x*. In other words, it is recommended to have at least 20 participants per group, and this number is increased as the observed *p*_extreme_ proportion deviates from 0.5. For two groups, the total sample size should be between 40 and 60. For five groups, the total sample size should be between 100 and 139.

As an example, in the previous illustration, there were four groups (requiring at least 4 × 20 = 80 total participants), and the most extreme observed proportion of 0.19 adds $50\;\times |\sin^{-1}\left(\sqrt{0.19}\right)-\sin^{-1}\left(\sqrt{0.5}\right)|\;=50\times 0.334=16.7186$ participants, for a recommended total sample size of 97. The total sample size in the illustration was actually 97, just the recommended sufficient number of participants. Also keep in mind, that as seen in the simulations, the groups should be approximately of equal size, with the ratio of the largest to the smallest group sizes being 1.5 or less.

## Two between-group factors

### An illustration

**Figure 3:**
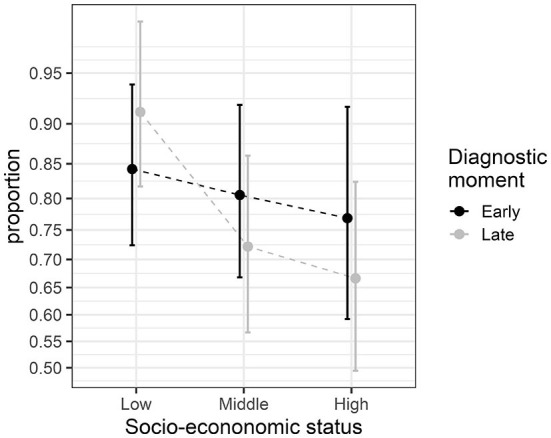
Proportions from the graduation study. The error bars show difference-adjusted 95% confidence intervals of the proportions (obtained with the package superb for R, Cousineau et al., 2021, as explained in the OSF website: osf.io/gja9h, folder *SecondIllustration*).

In this second section, we considered an examination of the proportions of young adults with dyslexia who graduate from college. The participants were divided based on whether they obtained an early (elementary school) or a late (high school) diagnosis of dyslexia and also on their socio-economic status (SES; low, medium, high). The design is therefore 2 × 3 with six independent groups. The observations are compiled in Table 3, and the observed proportions are illustrated in Figure 3. As seen in these data, low-SES participants tend to have better graduation rates. Although the difference-adjusted 95% confidence intervals are wide, the low-SES group as a whole looks distinct from the other two SES levels, something that will be confirmed formally in the upcoming two-way ANOPA.

**Table 3:** Graduation number (sample size between parentheses) of young adults with dyslexia based on diagnosis moment and socioeconomic status (SES).

	**Moment of diagnostic**					
	**Early**			**Late**		
**SES**	**Data**	***p***	***A***	**Data**	***p***	***A***
Low	75 (89)	0.8427	1.1591	84 (92)	0.9130	1.2656
Middle	62 (77)	0.8052	1.1100	52 (72)	0.7222	1.0131
High	40 (52)	0.7692	1.0652	42 (63)	0.6667	0.9532

Also shown are the proportions (*p*) and the Anscombe-transformed scores (*A*).

We began with the between-subject sum of squares using the groups' aggregates *A*_*ij*_ (the *i*th moment of diagnostic and the *j*th SES), which is given by:


(8) $$\begin{array}{l} {\text{SS}}_{\text{Between}}=\overset{p}{\underset{i=1}{\sum }}\overset{q}{\underset{j=1}{\sum }}{\left(A_{ij}-{\bar{A}}_{••}\right)}^{2}=\left(pq-1\right)\text{ var}\left(A_{ij}\right) \end{array}$$

where *p* = 2 and *q* = 3 in the illustration, where *Ā*_••_ is the grand mean across both factors, and where var(*A*_*ij*_) is the variance across the 6 Anscombe-transformed scores. The global variance is next broken down into Moment of diagnostic sum of squares (here labeled P), an SES sum of squares (here labeled Q), and an interaction sum of squares, labeled P × Q:


(9) $$\begin{array}{l} \;{\text{SS}}_{\text{P}}=\overset{p}{\underset{i=1}{\sum }}q{\left({\bar{A}}_{i•}-{\bar{A}}_{••}\right)}^{2}\\ \;=\left(\text{p}-1\right)q\text{ var}\left({\bar{A}}_{i•}\right)\\ \;{\text{SS}}_{\text{Q}}=\overset{q}{\underset{j-1}{\sum }}p{\left({\bar{A}}_{•j}-{\bar{A}}_{••}\right)}^{2}\\ \;=\left(q-1\right)p\text{ var}\left({\bar{A}}_{•j}\right)\\ {\text{SS}}_{\text{P}\times \text{Q}}=\overset{p}{\underset{i=1}{\sum }}\overset{q}{\underset{j=1}{\sum }}{\left(A_{ij}-{\bar{A}}_{i•}-{\bar{A}}_{•j}+\bar{\bar{A}}\right)}^{2}\\ \;={\text{SS}}_{\text{Between}}-{\text{SS}}_{\text{P}}-{\text{SS}}_{\text{Q}} \end{array}$$

in which ${\bar{A}}_{•j}=\frac{1}{p}\overset{p}{\underset{i=1}{\sum }}A_{ij}$ are the *q* marginal means across factor *P*, ${\bar{A}}_{i•}=\frac{1}{q}\overset{q}{\underset{j=1}{\sum }}A_{ij}$ are the *p* marginal means across factor Q, ${\bar{A}}_{••}=\frac{1}{pq}\overset{p}{\underset{i=1}{\sum }}\overset{q}{\underset{j=1}{\sum }}A_{ij}$ is the grand mean, and var(*Ā*_*i*•_) is the variance across the *p* means, whereas var(*Ā*_•*j*_) is the variance across the *q* means. From these and the degrees of freedom, we obtained the mean squares in the usual fashion:


(10) $$\begin{array}{l} \begin{array}{l} \;{\text{MS}}_{\text{P}}={\text{SS}}_{\text{P}}/\left(\text{p}-1\right)=q\text{ var}\left({\bar{A}}_{i•}\right)\\ \;{\text{MS}}_{\text{Q}}={\text{SS}}_{\text{Q}}/\left(q-1\right)=p\text{ var}\left({\bar{A}}_{•j}\right)\\ {\text{MS}}_{\text{P}\times \text{Q}}={\text{SS}}_{\text{P}\times \text{Q}}/\left(\left(\text{p}-1\right)\left(q-1\right)\right) \end{array} \end{array}$$

Regarding the error term, we again used the theoretical variance, assuming that *n*_*ij*_ is large enough (a number addressed in the simulation study below):


(11) $$\begin{array}{l} {\text{MS}}_{e}=\text{mean}({\text{var}}_{\text{th}}\left(A_{ij}\right))=\frac{1}{pq}\overset{p}{\underset{i=1}{\sum }}\overset{q}{\underset{j=1}{\sum }}\frac{1}{4\left(n_{ij}+\frac{1}{2}\right)} \end{array}$$

in which *n*_*ij*_ is the number of observations in cells *i, j* of the design. The sum of squares decomposition and the degrees of freedom for each term are illustrated with this schematic:


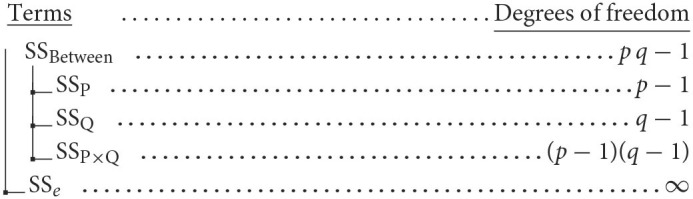


Test statistics are given by:


(12) $$\begin{array}{l} \begin{array}{l} \;F_{\text{P}}=\frac{{\text{MS}}_{\text{P}}}{{\text{MS}}_{e}}\\ \;F_{\text{Q}}=\frac{{\text{MS}}_{\text{Q}}}{{\text{MS}}_{e}}\\ F_{\text{P}\times \text{Q}}=\frac{{\text{MS}}_{\text{P}\times \text{Q}}}{{\text{MS}}_{e}} \end{array}\}\text{ or }\{\begin{array}{l} \;g_{\text{P}}=\left(\text{p}-1\right)\frac{{\text{MS}}_{\text{P}}}{{\text{MS}}_{e}}\\ \;g_{\text{Q}}=\left(q-1\right)\frac{{\text{MS}}_{\text{Q}}}{{\text{MS}}_{e}}\\ g_{\text{P}\times \text{Q}}=\left(\text{p}-1\right)\left(q-1\right)\frac{{\text{MS}}_{\text{P}\times \text{Q}}}{{\text{MS}}_{e}} \end{array} \end{array}$$

in either an *F* form (left) or a chi-square form (right). The chi-square form only requires the numerator degrees of freedom to assess its significance, whereas the *F* form has infinite degrees of freedom to the denominator.

The two-way ANOPA table is given in Table 4 along with Williams' corrections in the note (described below). As can be seen, factor SES has an effect [*F*_(2, ∞)_ = 6.39, *p* < 0.002], but factor Moment of diagnostic has no effect [*F*_(1, ∞)_ < 1], and there is no interaction between the two [*F*_(2, ∞)_ = 2.14, *p* = 0.12]. These results are in good agreement with the results depicted in Figure 3 when taking into account the difference-adjusted 95% confidence intervals.

**Table 4:** Two-way ANOPA table.

	**SS**	**df**	**MS**	***F***	***p*-value**
Between group	0.0611	5			
Moment (P)	0.0017	1	0.0017	0.5010	0.4791
SES (Q)	0.0445	2	0.0222	6.3949	0.0017
P × Q	0.0149	2	0.0074	2.1400	0.1177
Error	-	∞	0.0035		

Using William's correction for factor P, *c*_*P*_ = 1.0011, we obtain *F*_(1, ∞)_ = 0.5004, *p* = 0.4793, for factor Q, *c*_*Q*_ = 1.0015, we obtain *F*_(2, ∞)_ = 6.3853, *p* = 0.0017, and for factor P × Q, *c*_P × Q_ = 1.0066, we obtain *F*_(2, ∞)_ = 2.1261, *p* = 0.1193.

### Type I error rates with two between-subject factors

To examine type I error rates in two-way ANOPA, we chose to focus on the interaction effect. As in the previous section, we generated random samples. However, instead of varying the prevalence of the last group, we held it constant at ¾ and varied instead the number of levels of the second factor (*q*; 2, 3, or 4 levels). We explored total sample sizes larger than 50 only. There were 4 (number of levels of *p*) × 3 (number of levels of *q*) × 4 (population proportions π) × 11 (sample sizes *n*) = 528 conditions, with each of them replicated 500,000 times.

Williams's (1976) correction factor for an interaction term is given by


(6b) c=1+(p×q)2-16 n˜(p-1)(q-1)

where (*p* − 1) (*q* − 1) is the degrees of freedom of the examined effect, and *p* × *q* is the number of cells concerned by this correction.

In the population simulated, there is no true interaction effect P × Q and no true main effects of factors P and Q. Everything else is as in the previous simulation study described in Appendix A.

**Figure 4:**
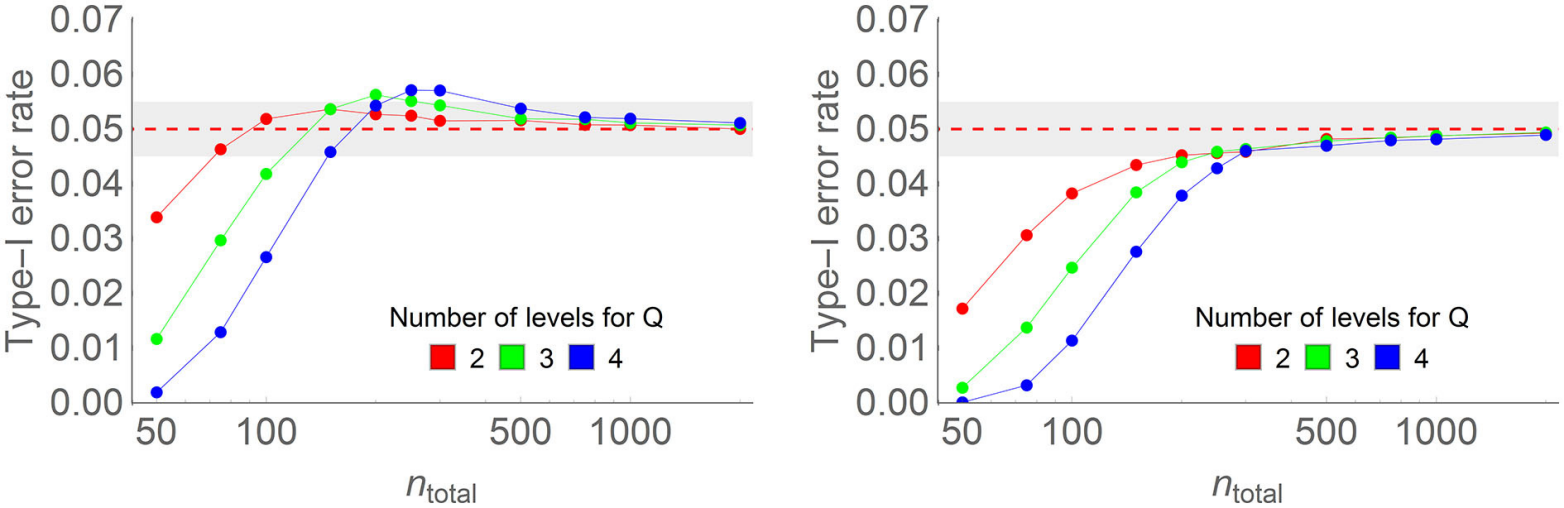
Type I error rate of the two-way ANOPA with **(right)** and without **(left)** the correction factor in some of the conditions of the full simulations (here, the number of levels of the *first* factor is 4, the population proportion is 0.3, the number of levels of the second factor ranges from 2 to 4 (colored lines), and sample sizes on the horizontal axis vary from 50 to 2,000 on a log scale. Each point is based on 500,000 simulations.

Figure 4 shows some results of the simulations without correction (left) and with the correction factor (right). The complete results are found in Appendix A. Again, without the correction factor, the type I error rates momentarily exceed α = 0.05, the chosen decision level, reaching up to 0.061 in some conditions shown in Appendix A Figure B1. The correction factor keeps the type I error rate below 0.0505 in all tested conditions; on the other hand, it accentuates the decrease of the type I error rate below the nominal alpha level for smaller samples, reducing the chance of detecting real significance (i.e., the test is more conservative for small samples).

The total sample size sufficient to reach the theoretical variance is larger, but there are also many more groups (*p* × *q* groups) than in the previous study. The rule of thumb of Equation 7 is still roughly valid to provide a sufficient sample size per group in a two-way design.

## A within-subject design

### An illustration

As a third illustration, we considered a situation in which 30 participants receive one of three drugs and a placebo in a random order to see if they are experiencing a period of delirium or not (“1” here means that at least one event is happening).

**Figure 5:**
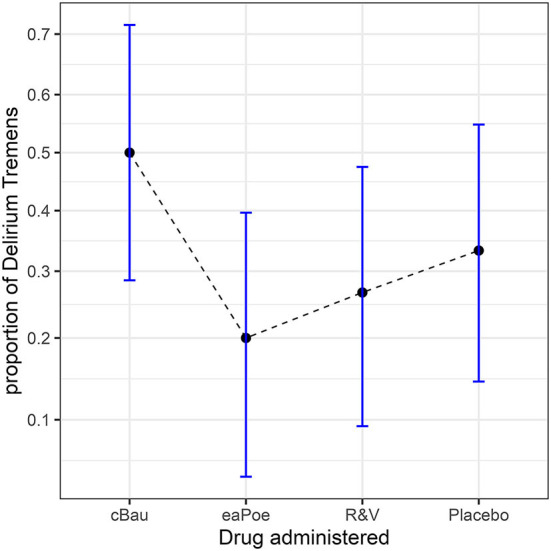
Proportions from the study looking at the effects of three drugs on the presence of delirium episodes. The error bars show correlation- and difference-adjusted 95% confidence intervals (obtained with the package superb for R, Cousineau et al., 2021, as explained in the OSF website osf.io/gja9h, folder *ThirdIllustration*).

The results are shown in Figure 5 along with correlation- and difference-adjusted 95% confidence intervals (more on these error bars in the last section). The results seem to indicate that the cBau drug favors the presence of at least one episode of delirium, affecting 50% of the participants, whereas it does not exceed 35% with the other drugs.

This study uses a within-subjects design, in which individual data or events are generally correlated. For instance, some participants may be more susceptible to the syndrome, irrespective of the drug taken. To estimate the amount of correlation, it is necessary to have access to the raw data coded with “0s” and “1s:” these are given in Table A1 available on the OSF site, osf.io/gja9h/, folder *ThirdIllustration*.

Correlation (at least when it is positive) is beneficial to statistical inference because it helps subtract a substantial portion of the between-subject variance from the between-condition error term, thus increasing statistical power. Remember that the reliability of repeated measures increases with the number of such measures, as embodied by the Spearman-Brown prophecy formula (Anastasi and Urbina, 1997). As an example, the definition of Cronbach's (1951) famous internal consistency index must be considered. Also called coefficient alpha (α, not to be confused with the decision threshold α), it is


(13) $$\begin{array}{l} \alpha =\frac{k}{k-1}\left(1-\frac{S}{V}\right) \end{array}$$

where *V* = var(*x*_•*j*_), the variance of the *n* sums of responses, one sum per participant, with $x_{•j}=\overset{k}{\underset{i=1}{\sum }}x_{ij}$, and *S* = $\overset{n}{\underset{j=1}{\sum }}\text{var}\left(x_{i•}\right)$ the sum of the *k* individual variances, with $x_{i•}=\overset{n}{\underset{j=1}{\sum }}x_{ij}$ in which *x*_*ij*_ denotes the *i*th score (success or failure, 1 to *k*) of the *j*th participant (1 to *n*). Herein, *k* is the number of repeated measures (*k* is akin to the number of levels *p* in the between-group design). As the number of items grows, *V* increases more rapidly than *S*, owing to the positive correlations between measures across participants, and the ratio 1 − *S*/*V* tends toward 1. Let the symbol α_1_ denote the mean variance-weighted correlation coefficient between measures. The Spearman-Brown formula links the two quantities through the formula:


(14) $$\begin{array}{l} \alpha =\frac{k\alpha_{1}}{1+\left(k-1\right)\alpha_{1}} \end{array}$$

in which α_1_ (named unitary α in Laurencelle, 1998) can be seen as the contribution of a single item to the overall consistency of the set. In the very restricted case where all items' variances are equal, then α_1_ equals $\bar{r}$, the average Pearson correlation between the pairs of items (proof is given in deVellis, 2003, p. 36; also see Cousineau, 2019).

In the most general case, by isolating α_1_ in Equation (14), i.e., α_1_ = α/(*k* − (*k* − 1)α), and inserting Equation (13), we obtained the general expression for α_1_:


(15) $$\begin{array}{l} \alpha_{1}=\frac{V-S}{\left(k-1\right)S} \end{array}$$

This measure is a general measure of correlation in designs with repeated measures (Laurencelle, 1998; Goulet-Pelletier and Cousineau, 2019).

From these considerations, we can see that the error variance can be partitioned according to α_1_ into an unsystematic part (1 − α_1_) and a participant-related part (α_1_). Removing the latter part, we obtain a purer, smaller, and more specific estimate of error variance.

Returning to the ANOPA based on a one-way, repeated-measure design, we use the following formulas:


(16) $$\begin{array}{l} \begin{array}{l} \;{\text{SS}}_{\text{P}}=\overset{k}{\underset{i=1}{\sum }}{\left(A_{i}-\bar{A}\right)}^{2}=\left(k-1\right)\text{ var}\left(A_{i}\right)\\ {\text{MS}}_{\text{P}}={\text{SS}}_{\text{P}}/\left(k-1\right)=\text{ var}\left(A_{i}\right)\\ \;{\text{SS}}_{e}=\left(1-\alpha_{1}\right)\overset{k}{\underset{i=1}{\sum }}{\text{ var}}_{\text{th}}\left(A_{i}\right)=\overset{k}{\underset{i=1}{\sum }}\frac{1-\alpha_{1}}{4\left(n+\frac{1}{2}\right)}\\ {\text{MS}}_{e}={\text{SS}}_{e}/k=\left(1-\alpha_{1}\right)\text{mean}({\text{var}}_{\text{th}}\left(A_{i}\right))=\frac{1-\alpha_{1}}{4\left(n+\frac{1}{2}\right)} \end{array} \end{array}$$

in which *A*_*i*_ denotes Anscombe-transformed scores *A*(*x*_*i*_, *n*) for the *i*th measurement, $\bar{A}$ denotes the mean score over all the *A*_*i*_, var(*A*_*i*_) is the variance across the *p*-transformed scores, SS is the sum of squares, MS is the mean square, and *n* is the number of observations in the different conditions (all equal in a repeated-measure design). Finally, as before,


$$\begin{array}{l} F_{\text{P}}=\frac{{\text{MS}}_{\text{P}}}{{\text{MS}}_{e}}\text{ or }g_{\text{P}}=\left(k-1\right)\frac{{\text{MS}}_{\text{P}}}{{\text{MS}}_{e}} \end{array}$$

whereas, *F*_*P*_ follows an *F* distribution with *k*−1, ∞ degrees of freedom, *g*_*P*_ follows a chi-square distribution with *k*−1 degrees of freedom.

Returning to the example, Table 5 shows the aggregated scores. The variance of the *x*_•*j*_ totals, *V*, is 1.5966, and the sum of the *k* variances, *S*, is 0.8563, yielding an α_1_ of 0.2881. Table 6 shows the one-way ANOPA for repeated measures. The result is significant (*p*-value of 0.0276). When the correction factor (Equation 6a) is used, the *p*-value increases to 0.0284.

**Table 5:** Number of successes for the three measurements in the drug study for delirium treatment (*n* = 30).

	**Number of success (*s*)**	***p* = *s*/*n***	** $A=\sin^{-1}\left(\sqrt{\frac{s+\frac{3}{8}}{n+\frac{3}{4}}}\right)$ **
cBeau	15	0.5000	0.7854
eaPoe	6	0.2000	0.4727
R&V	8	0.2667	0.5491
Placebo	10	0.3333	0.6198
Variance			0.0178

Also shown are proportions and Anscombe-transformed scores (A).

**Table 6:** One-way within-subject ANOPA table.

	**SS**	**df**	**MS**	***F***	***p*-value**
Treatment (P)	0.0534	3	0.0178	3.043	0.0276
Error	-	∞	0.0058		

The correlation α_1_ is 0.2881. Using the correction factor *c*_P_ = 1.0069, we obtain *F*(3, ∞) = 3.0217, *p* = 0.0284.

To summarize this third illustration, we found a significant difference [*F*_(3, ∞)_ = 3.043, *p* = 0.0276] between conditions, with or without the correction factor. *Post-hoc* HSD tests performed on OSF, folder *ThirdIllustration*, suggest that cBau and eaPoe are the only two conditions with a significant difference (Tukey's HSD *q* = 4.0888, *p* = 0.0200, here computed without the correction factor, or *q* = 4.0747, *p* = 0.0225 with the correction factor; note that the square root of the correction factor must be used in *post-hoc* HSD tests).

### Type I error rate in within-subject designs

**Figure 6:**
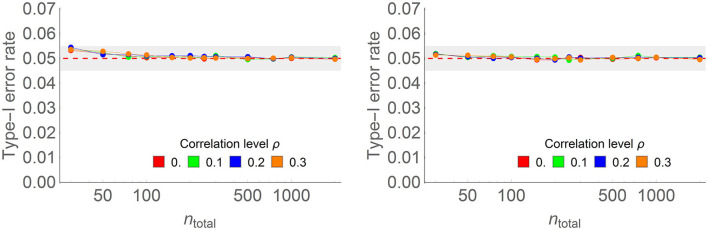
Type I error rate of the one-way within-subject ANOPA with **(right)** and without **(left)** the correction factor in some of the conditions of the full simulations (here, the number of measurements is 4, the population proportion is 0.3, the population correlation ρ ranges from 0.0 to 0.3 (colored lines), and sample sizes on the horizontal axis vary from 20 to 2,000 on a log scale. Each point is based on 150,000 simulations.

We did a final Monte Carlo study to examine the behavior of the type I error rates in within-subject designs as a function of the amount of correlation between the items. We ran the simulation first without and then with the correction factor. In every simulation, we estimated α_1_ from the data. Appendix A provides additional details. Figure 6 shows examples of the results, whereas the complete results are given in Appendix A.

As seen before, the test is conservative when the sample size is not sufficiently large. When two measurements are used, the simulations show small areas where type I error rates exceed 0.05 (reaching 5.8% when there are 30 simulated participants), suggesting that the correction factor is not entirely adequate in this design. Except when there are two measurements, the results are little influenced by their number. This implies that the sufficient sample size to reach theoretical variance is based on the number of independent groups (here only one), not the number of levels of the factor. As the simulations reported had 30 or more participants, meeting the 20-participants-per-group rule of thumb, it explains why the areas where the type I error rate exceeds 0.05 are small.

The correction factor is effective in eliminating the excess above 0.05 only when there are more than two measurements; with two measurements, there is no visible difference with or without the correction factor.

## Statistical power analyses

In planning a new study, it is customarily recommended to estimate the sample size required to optimize the odds of detecting an effect if there really is one (Laurencelle, 2007; Brysbaert, 2019). To that end, the usual steps are: (i) get access to relevant published information and determine the expected results; (ii) convert them into a standardized effect size; (iii) set the decision threshold and desired power (conventionally labeled α and 1 – β), from which the needed sample size is mathematically deduced.

In ANOVA, the effect size generally used is *f*^**2**^, the ratio of the variance of the effect to the error variance (or its square root, *f;* Cohen, 1988, 1992). If prior results or a meta-analysis can suggest the magnitude of the population effect size, then the power analysis must be performed on that estimate.

The same process can be followed with ANOPA. As before, the variance induced by the effect must be computed after the expected proportion values have been Anscombe-transformed. Then, this variance is divided by the harmonic mean of the planned number of participants per group. Also, because the expected effect variance is stipulated as a population variance (rather than a sample variance), its divisor is the number *p* of conditions, not *p* – 1.

For example, let us suppose that a replication of the study on incubation (first illustration) is to be performed. Let us assume that the expected proportion corrects for the replications (rounding the results of the first illustration) are 0.32, 0.64, 0.40, and 0.16.

The variance of the effect, noted $\sigma_{\text{P}}^{2}$, is the putative population variance between the four Anscombe-transformed scores, that is, 0.0302. The same result is obtained by computing the sample variance on the Anscombe-transformed proportions var(*A*_*i*_) and multiplying it by (*p* − 1)/*p* to make it a population variance, where *p* is the number of levels considered, in this case, four.

Regarding the error variance, noted $\sigma_{e}^{2}$, it is *ñ* × 1/(4*ñ*) when the additive term $\frac{1}{2}$ is considered negligible. Taken together,


(17) $$\begin{array}{l} f^{2}=\frac{\sigma_{\text{P}}^{2}}{\sigma_{e}^{2}}\approx \frac{\frac{\text{p}-1}{p}\text{ var}\left(A_{i}\right)}{\tilde{n}\times 1/\left(4\tilde{n}\right)}=4\frac{\text{p}-1}{p}\text{ var}\left(A_{i}\right) \end{array}$$

which is independent of the sample size in need of planning. In the present example, *f*^2^ = 0.1281 and thus, *f* = $\sqrt{0.1281}$ = 0.3579. According to Cohen's guidelines (Cohen, 1992), this is a moderate-to-large effect size. Entering f, α, and 1 − β into a power computation tool such as G*Power (Mayr et al., 2007), we obtained the recommended sample size. With the following *f* = 0.3579, α = 0.05, and 1 − β = 0.80, we were invited to recruit 92 participants (the exact number is actually 89.13 but as G*Power assumes equal group sizes, the result was rounded up to the nearest integer participant within each group; also, in a priori power computation with G*Power, it is not possible to set the denominator degrees of freedom to ∞; doing so would have reduced the number of participants needed to 88).

Alternatively, the non-centrality parameter λ required for the power calculation can be computed directly using $\lambda =n_{\text{total}}\times f^{\text{2}}$ which, assuming a sample size of 100, equals 12.8076. With this λ, α = 0.05, a numerator degree of freedom of 3 and a large number for the denominator degrees of freedom, G^*^Power indicates a power of 86.53% (under *F*-tests: Generic *F*-test).

As a validation, we estimated power by running a simulation with the population proportions set to 0.32, 0.64, 0.40, and 0.16 (noted above), and 100 simulated participants. We found a rejection of the null hypothesis in 87.5% of the simulations over 1,000,000 replications, supporting the power analysis conclusion. The simulation used the correction factor; without it, we found 87.7% rejection of the null hypothesis (with both standard errors of the simulation results being smaller than 0.1%).

As a final note, it must be remembered that the sufficient sample size *n*_sufficient_ (e.g., Equation 7) to reach theoretical variance must also be met so that, when planning a study, the largest of both analyses (power analysis and sufficient sample size) must be retained. Here, Equation 7 imposes a minimum of 99 participants, with the most different prediction from 0.50 being 0.16.

## Confidence intervals and plots of adjusted confidence intervals

More and more, researchers are invited to present their results graphically. One reason for this is to take some distance from the dichotomized interpretation of results introduced by decision thresholds (significant vs. non-significant; Trafimow et al., 2018; Amhrein et al., 2019). Another reason is to be more sensitive to trends and patterns of results that are not easily apparent from dichotomized decisions based on *p*-values (Loftus, 1993). To be meaningful, aggregates and other summary statistics must be presented with accompanying measures of imprecision, such as standard errors or confidence intervals, among others. In this section, we show how standard errors and confidence intervals can be obtained for proportions (extending Hogg and Craig, 1978).

The standard error is a measure of the variability or uncertainty of a summary statistic. For a given Anscombe-transformed aggregate, the standard error is theoretically given by the square root of the variance, ${\text{var}}_{\text{th}}\left(A\left(s,n\right)\right)=1/\left(4\left(n+\frac{1}{2}\right)\right)$, so that standard error in the *i*th group for Anscombe-transformed proportions is


$$\begin{array}{l} SE_{i}=\frac{0.5}{\sqrt{n_{i}+\frac{1}{2}}}; \end{array}$$

it depends only on sample size, so larger samples give more precise estimates, with narrower confidence intervals.

The *g* test statistics, $\left(\text{p}-1\right)F=\left(\text{p}-1\right)\overset{p}{\underset{i=1}{\sum }}{\left(A_{i}-Ā\right)}^{2}/\left(\left(\text{p}-1\right)MS_{e}\right)$, is, under homogeneity of variances, equal to


$$\begin{array}{l} \overset{p}{\underset{i=1}{\sum }}{\left(\frac{A_{i}-Ā}{SE_{i}}\right)}^{2} \end{array}$$

This is a sum of squared z-scores. Both assumptions (homogeneity of variance and normality of the distribution) are actually guaranteed for Anscombe-transformed scores with a sufficient *n*. Hence, the Gaussian *z* distribution can be used to get γ × 100% confidence interval limits as:


(18) $$\begin{array}{l} \left[A+z_{\frac{1}{2}-\frac{\gamma }{2}}\times SE_{A},A+z_{\frac{1}{2}+\frac{\gamma }{2}}\times SE_{A}\right] \end{array}$$

Chen (1990) and Lehman and Loh (1990) examined the behavior of approximate confidence intervals for proportions. As proportions are discrete (with a sample of size 10, for example, observed proportions could be 0.0, 0.1, 0.2, et cetera, but cannot achieve intermediate values), these authors showed that the most accurate confidence intervals will actually sometimes be a little too liberal, and sometimes a little too conservative. Chen (1990) showed that arcsine-based confidence intervals have almost the least varying coverage rates and suggested replacing the weights 3/8 and 3/4 with other weights. However, the reduction in coverage variability is immaterial. In unreported simulations, coverage of the 95% confidence interval given in (18) never went below 94.2% and never exceeded 95.8%. An alternative method to get confidence intervals is based on the pivot method of (see Clopper and Pearson, 1934; Leemis and Trivedi, 1996). This technique's coverage for a 95% confidence interval will never be below 95% but can be as conservative as 98%.

Equation (18) defines a *stand-alone* confidence interval because it is to be used when a result is compared to a pre-specified value of interest. However, the whole point of ANOPA is to compare results with each other, not to fix values. Consequently, we need confidence intervals that allow for comparing results.

The difference-adjusted confidence interval is the adequate interval when comparing independent groups and interpreting their mutual differences. To obtain it, the interval width in Equation 18 is multiplied by $\sqrt{2}=1.41$, which makes it 41% longer. Figures 1, 3 were made with difference-adjusted confidence intervals. One way to interpret these intervals is to look for inclusion: if one result is included in the adjusted confidence interval of another result, these two results are probably not statistically different at the γ level, typically 95% (Cousineau, 2017).

In within-subject designs, knowledge about the correlation between repeated measures can be used to improve the precision of results. Many techniques have been developed that account for the presence of correlation in repeated measures designs, notably Loftus and Masson (1994), and Cousineau-Morey (Cousineau, 2005; Morey, 2008). However, all these techniques boil down to a multiplication of the standard error value by $\sqrt{2\left(1-r\right)}$ (Cousineau, 2019), with *r* being a measure of correlation (we use α_1_ in the context of ANOPA). Thus, the stronger the correlation, the shorter the error bars. The correlation-and difference-adjusted confidence intervals implement this technique, allowing the comparison of correlated measurements with each other. Figure 5 was made with those confidence intervals. Cousineau et al. (2021) demonstrated the mathematical validity of these adjusted confidence intervals with respect to statistical inference.

The Anscombe-transformed scores and their adjusted confidence intervals can be plotted directly. However, the typical reader might prefer to see proportions rather than Anscombe-transformed scores on the vertical axis. In that case, it is possible to compute the transformed scores interval limits, then reverse the transformation so that a plot of proportions can be made. This is what was done in Figures 1, 3, 5. A simple way to un-transform the confidence limits is with (sin(limit))^2^. A more accurate way, which takes into consideration the terms 3/8 and 3/4, is found on the OSF website, although the difference is barely discernible. Finally, it should be noted that in Figures 1, 3, 5, we did not use a linear scale for the vertical axis but an arcsine scale. This is mostly visible in Figure 3: the intervals around 0.5 are narrower than the intervals around 0.9. This scale is showing Anscombe-transformed scores linearly, so inverting the transformation on the confidence limits does not introduce distortions.

The R package “superb” can be used to get such plots (Cousineau et al., 2021) as explained on https://dcousin3.github.io/superb/articles/VignetteC.html. With “superb” the adjusted intervals are obtained by adding options so that no programming is required.

## Conclusion

ANOPA is a complete series of analyses for proportions, applicable to any design. It is based on Anscombe's (1948) arcsine transformation, whose error variance is theoretically known. To that end, the sample must be of sufficient size; an approximate rule was given to estimate the sufficient sample size. ANOPA is based on a few assumptions: that the measures are binary, and the effects on the Anscombe-transformed scores are additive. Sampling must be done as with ANOVAs: the participants are drawn independently of each other. There is no normality assumption and no homogeneity of variance assumption in ANOPA; these are built into the arcsine transform.

Simulations indicate that the groups have to be roughly equal in size (a ratio between the largest and the smallest group up to 1.5 to 1 being acceptable); also, the proportions tested should not be too extreme when the sample is small. In these circumstances, ANOPA is never liberal, that is, its type I error rate never exceeds the decision threshold, making this test very reliable.

In this text, we provided examples of a one-way between-subject design (first illustration), a two-way between-subject design (second illustration), and a one-way repeated-measure design (third illustration). Interested readers will find on the OSF website, https://osf.io/gja9h/ (folder *MixedDesign*), a fourth example illustrating a mixed (one-factor within-subject and one-factor between-subject) design based on unpublished research data from G. Trudel and her colleagues. In a fifth example, how to perform simple effects analyses in a two-way design following a significant interaction is shown (OSF website, folder *LogisticRegression*; the ANOPA analysis is compared to logistic regression in that example). These examples should help the researchers test more complex designs.

The analyses without correction factors sometimes show type I error rates exceeding the decision threshold, however, deviations are small (never exceeding 6.3% for a 5% decision threshold); the worst cases are found with five groups (we did not simulate more groups) in one-way ANOPA. A simple correction factor has been proposed, which has been shown to be very effective for un-extreme population proportions (within 0.2 and 0.8): in these simulations, the type I error rate never exceeded the 5% threshold by more than 0.4%. The correction factor was mathematically derived by Williams (1976).

Warton and Hui (2011) examined similar questions and found that for extremely tiny samples (two groups of 3 or two groups of 6), a logistic regression analysis had more chances to detect an effect. This is not unexpected, as ANOPA is very conservative for small samples (and logistic regression is very liberal). Yet this advantage is for sample sizes for which using statistical testing is quite dubious.^1^ In Appendix B on OSF at https://osf.io/gja9h/, folder *Appendices*, we review many limitations of the logistic regression analyses and how ANOPA addresses these limitations while offering many more additional analyses (allowing the detection of main effects, interaction effects, simple effects, post-hoc tests, orthogonal contrasts tests, and et cetera).

With the capabilities to assess significance, plan statistical power, and make informative plots, proportions are now just another regular dependent variable in the researchers toolkit.

## Data availability statement

The original contributions presented in the study are included in the article/Supplementary material, further inquiries can be directed to the corresponding author.

## Author contributions

Both authors listed have made a substantial, direct, and intellectual contribution to the work and approved it for publication.

## Footnotes

^1^With three subjects per group, there are only 64 possible outcomes. ANOPA detects as significant only the two samples in which all the participants in one group failed the measure and all the participants in the other passed it. The logistic regression also detects as significant the 12 outcomes in which one of the groups has two-thirds of failures compared to another group where all are successes (or vice versa).
