# Supplementary material for: Analysis of proportions using arcsine transform with any experimental design
Source: Front Psychol. 2023 Jan 30;13:1045436. doi: 10.3389/fpsyg.2022.1045436 (PMC9922716; doi:10.3389/fpsyg.2022.1045436)
Supplement: Supplementary file 1 [file Presentation_1.pdf]

ANALYSIS OF PROPORTIONS USING ARCSINE TRANSFORM WITH ANY EXPERIMENTAL DESIGN

Louis Laurencelle Denis Cousineau

Université du Québec à Trois-Rivières *Université d'Ottawa*

Published in Frontiers in Psychology.

Running head: ANOPA

Address correspondence to Denis Cousineau, École de psychologie, Université d'Ottawa, 136 Jean-Jacques Lussier, K1N 6N5 Ottawa, Canada. Phone: (613) 562-5800 #7910; E-mail: denis.cousineau@uottawa.ca; ORCID: (DC) 0000-0001-5908-0402

Appendix A: Type I Error Rate Of ANOPA Estimated From Simulations

Simulations were performed using the open-access Wolfram Language and are available on the OSF site https://osf.io/gja9h/, folder *TypeI-EstimationSimulations*. Unless specified otherwise, each simulation iterated 500,000 times in each condition (described below). We chose this number so that the 95% confidence interval of a single simulation is small (0.1%); this was necessary as most of the deviations to the nominal alpha level were well within 0.5%. In the Figures presenting the simulation results, a shaded area covering ± 0.5% is added as a reference. For each simulated dataset, we recorded whether the test rejected the null hypothesis that all the proportions are equal or not. As all the proportions of successes are equal at the population level, a rejection decision is a type I error. All tests were performed at the decision threshold α of .05. All the simulations examined population proportions between 0 and 0.5; the results are all identical for population proportions between 0.5 and 1 (i. e., 0.1 and 0.9 return the same results).

One-way ANOPA

Methodology. The random variates were generated from a multinomial distribution. This distribution requires the population proportions of successes per condition but also the population proportions of failures. To generate these, we proceeded as follow: One vector of length p is constructed, equal to

$$\left(\frac{\psi}{(p-1)+\psi} \quad \underbrace{\frac{1}{(p-1)+\psi} \quad \cdots \quad \frac{1}{(p-1)+\psi}}_{p-1 \text{ times}}\right).$$

This vector contains equal population proportions for all groups except the first. This first group has a population proportion multiplied by its prevalence relative to the other groups (noted ψ herein; either same, 1.5 or 2 times more prevalent, or ¾ or ½ less prevalent). Finally, the vector of population proportion of successes is obtained by multiplying the above vector by the population rate of success, π, and the vector of population proportion of failures, by multiplying it by the population rate of failure, $1 - \pi$. These two vectors are joined into a vector of length $2\,p$. From this model, n data are sampled, the successes in the first p columns, and the failures in the last p columns.

Note that it occasionally happened that no success or no failure was observed in a group. For the determination of the type I error rate with uncorrected test statistics, this has no influence as the Anscombe transformation can be computed even when the number of cases are 0. With the correction factor, which uses the harmonic mean, it was necessary to handle these cases because the harmonic mean is undefined when one cell is zero. In the reported simulations, these rare occurrences were re-run. In an unreported set of simulations, we instead computed the harmonic mean only from the non-empty cells. Both sets of results were undistinguishable.

The conditions are: number of groups (p; 2 to 5; 4 levels); true population rate of success (π; 0.1, 0.2, 0.3, and 0.5; four levels); prevalence of the first group relative to the other groups (ψ; ½, ¾, 1, 1.5, 2; five levels); total sample size (n; 30, 50, 75, 100, 150, 200, 250, 300, 500, 750, 1000, 2000; 12 levels), for a total of $4 \times 4 \times 5 \times 12 = 960$ conditions. Each simulation was replicated 500,000 times in a given condition; they were run a second time with the correction factor.

Because we were also interested in finding what sample size is enough to reach the theoretical variance, we extrapolated the results upward to find the sample size whose type I error rate would exceed 4.5%.

Results. Figure OSFA1 shows all the results (the figures in this appendix are available as supplementary material on the OSF web site, https://osf.io/gja9h/, folder *AppendixFigures*). As seen, when total sample size is small, the one-way ANOPA is conservative. With two groups, it reaches the nominal α when the total sample size is between 30 and 75 (based on the population parameter, proportions closer to 0.5 requiring fewer participants); for 5 groups, it takes between 35 and 200 participants to reach the nominal α.

Without correction, the type I error rates momentarily overshoot the target 5%, reaching 6.4% in some conditions. Figure OSFA2 shows the results when the correction factor is applied to the test statistic. As seen, overshooting disappears almost entirely except for an extreme population proportion of 0.1. Sufficient sample sizes to reach 4.5% or above type I error rates are between 75 and 150 for 2 groups and between 150 and 250 for 5 groups.

See Figures OSFA1 and OSFA2 on OSF site https://osf.io/gja9h/ folder *AppendixFigures*

Two-Way Between-Subject ANOPA

This simulation followed the above methodology with only three differences: the prevalence factor was held fix at 0.75; the factor "Number of levels for the second factor" was introduced, with 3 levels (2, 3 and 4); and the smallest sample size, 30, was removed.

The complete results are seen in Figures OSFB1 (no correction factor) and OSFB2 (using the correction factor).

See Figures OSFB1 and OSFB2 on OSF site https://osf.io/gja9h/ folder *AppendixFigures*

One-Way Within-Subject ANOPA

Methodology. The study of type I error rates in the repeated measure design was performed in a similar fashion to the one used in one-way ANOPA except for the following differences. First, the factor prevalence present in the first study was removed; prevalence is irrelevant here as there is only one group of participants. It was replaced by a factor *correlation* between the scores (ρ; 0.0, 0.1, 0.2, 0.3; 4 levels). The 0.0 correlation level actually reproduces the result of the first simulation study with equal prevalence in all groups. The other factors manipulated are: the number of measurements (p; from 2 to 5; 4 levels), the proportion of "1"s in the population (π; 0.1, 0.2, 0.3, or 0.5; 4 levels), and the sample size (n; 30, 50, 75, 100, 150, 200, 250, 300, 500, 750, 1000 and 2000; 12 levels) for a total of 4 × 4 × 4 × 12 = 768 conditions.

Second, to generate correlated binary scores, we used the method of Lunn and Davis (1998). It can only produce scores with an equal population proportion, but this is what is needed in the present study, as we investigate the null hypothesis that all proportions are equal. This method generates raw scores from which the mean correlation is estimated using α_1 (the results were undistinguishable from those performed with the $\bar{r}$ and r_W estimators; Cousineau & Goulet-Pelletier, 2021) after which the number of successes are compiled and the analysis is continued as in Study 1 with the only provision that the error term is multiplied by 1 minus α_1. Finally, generating raw data

being considerably more memory intensive, we reduced the number of replications to 250,000.

Results. The results are shown in Figure OSFC1 for the unadjusted rejections. In Figure OSFC2, the test statistics are adjusted using the correction factor.

See Figures OSFC1 and OSFC2 on OSF site https://osf.io/gja9h/ folder *AppendixFigures*

References

Cousineau, Denis, Goulet-Pelletier, Jean-Christophe (2021) A study of confidence intervals for Cohen's dp in within-subject designs with new proposals, *The Quantitative Methods for Psychology*, *17*(1), 51-75. doi: 10.20982/tqmp.17.1.p051

Lunn, A. D., Davies, S. J. (1998). A note on generating correlated binary variables. *Biometrika*, *85*, 487-490. doi: 10.1093/biomet/85.2.487
