# Supplementary material for: Analysis of proportions using arcsine transform with any experimental design
Source: Front Psychol. 2023 Jan 30;13:1045436. doi: 10.3389/fpsyg.2022.1045436 (PMC9922716; doi:10.3389/fpsyg.2022.1045436)
Supplement: Supplementary file 2 [file Presentation_2.pdf]

ANALYSIS OF PROPORTIONS USING ARCSINE TRANSFORM WITH ANY EXPERIMENTAL DESIGN

Louis Laurencelle Denis Cousineau

Université du Québec à Trois-Rivières *Université d'Ottawa*

Published in Frontiers in Psychology.

Running head: ANOPA

Address correspondence to Denis Cousineau, École de psychologie, Université d'Ottawa, 136 Jean-Jacques Lussier, K1N 6N5 Ottawa, Canada. Phone: (613) 562-5800 #7910; E-mail: denis.cousineau@uottawa.ca; ORCID: (DC) 0000-0001-5908-0402

Appendix B: Using logistic regression instead of ANOPA

Some readers will notice that logistic regression models are also used to analyze proportions. However, we believe that this analysis has a few shortcomings when it is applied to proportions.

First, the type-I error rate is more liberal than the nominal α, reaching type-I error rates as high as 8% in many scenarios (where it never exceeded 6.4% for the uncorrected ANOPA and never exceeded 5.4% for the corrected ANOPA). To assess these type-I error rates, we redid the first simulation described in Appendix A using a logistic regression analyses instead of an ANOPA. The only difference is that only 20,000 simulated data sets per point are tested; the logistic regression, being an iterative optimization problem, takes far more time to perform than the ANOPA. See the Figure FigureOSFD1.png (on the OSF site, folder *AppendixFigures*). As seen, sample sizes have to exceed 250 before the type-I error rate returns to the nominal α. When the true population proportion is low (0.1), samples have to be in the thousands before type-I error rate returns to the nominal α. This undesirable behavior is not unexpected. The primary reason is that logistic regression uses the logit transformation rather than the Anscombe transformation. This transformation is not variance-stabilizing (as documented in Laurencelle, 2021b). Actually, the variance of the logit-transformed scores is reciprocal of the proportions, exacerbating the homogeneity of variances assumptions used in regression models. Also, the test is based on Wilks (1938)'s likelihood ratio test which is a slowly asymptotic test (*t* tests and *F* tests refines Wilks tests to small samples).

We also believe that logistic regression lacks statistical power in some situations. Consider as an example the second illustration of the main text. A logistic regression finds no significant coefficients. The coefficients estimated by the analysis are given in Table B1. Twice the log-likelihood index of fit is 429.22. If we constraint the model to no interaction terms, twice the log-likelihood index of fit increases by 4.57 which is a non-significant increase according to a likelihood ratio test (Wilks, 1938). This indicates that the data have no detectable differences when a logistic regression approach is used where -by contrast- ANOPA sees a main effect (see the main text).

Insert Table B1 about here

Another reason for this reduced statistical power is also easy to identify: The coefficients are not orthogonal because a non-linear transformation is used (using structural equation modeling, covariances between the coefficients could be assessed). These artificially-induced inter-correlations between the coefficients may create spurious effects through multicolinearity, something totally un-addressed when logistic regressions are used to analyze proportions. In ANOPA, the decomposition of the total effect into additive components guarantees its orthogonality.

Second, the logistic regression does not test effects (main effects, interactions, etc.), it tests coefficients one by one. As seen in Table B1, there are 6 coefficients in a 2 × 3 design (a saturated model). The intercept is the proportion in the reference condition (here, Early diagnostic + Low SES). All the other observed scores are predicted from various combinations of the coefficients. In Table B1, the two interactions coefficients

are close to significant. Yet, they say nothing regarding the presence or absence of an interaction effect which is the true sorts of question an analyst has.

Third, the logistic regression is sensitive to how the factors are coded. One level for each factor must be a level zero condition; this cell serve as a baseline for all the other estimates. However, if you change which level is the level zero cell, you get completely different estimates. Consider a dataset similar to the second illustration but where two cell scores are lowered to have significant effects: The resulting data are seen in Table B2. The scores are also illustrated in Figure B1.png found on the OSF site osf.io/gja9h/ folder *LogisticRegression*.

Insert Table B2 about here

When SES Low is coded as zero (and SES-Middle as 1), the logistic regression returns two interaction coefficients that are significantly different from zero. However, when SES middle is coded as zero (and SES low as 1), then the main effect of Moment of diagnostic becomes significant (along with the second interaction). The two analyses are summarized in Table B3.

Insert Table B3 about here

Seeing a main effect coefficient appear or disappear based on coding is unexpected to ANOVA users (but is expected by the fact that logistic regression does not test effects, as said above). The lower part of Table B3 shows the ANOPA results with either coding; as seen, they are totally identical, indicating a pregnant interaction effect.

The data and analyses for this section are available on the OSF site, https://osf.io/gja9h/ folder *LogisticRegression*.

By contrasts, ANOPA is testing effects (not coefficients); is unaffected by coding; finally, the effects are orthogonal, as demonstrated by their additivity so that spurious effects, multicolinearity and consequently reduced statistical power are impossible with ANOPA.

One additional demonstration of the additivity of ANOPA is that the interaction seen on the middle part of Table B3 can be decomposed into simple effects as is commonly done in ANOVA analyses. The results are seen in the bottom part of Table B3. The two simple effects' sums of squares (0.0444 and 0.1289) totalize 0.1333, which is indeed a valid decomposition of the interaction and the Moment of Diagnostic effects (whose sum of squares, 0.0442 and 0.0890, do totalize 0.1332; the difference being caused by rounding errors). We clearly see that SES has an effect only for late diagnostic participants. This result is not apparent for logistic regression users.

Some more advantages are a) that the ANOPA can be used when the rate of success in one or some cells is 0% or 100%; whereas the logit transformed is undefined in these situations; b) the ANOPA lead to an easy-to-compute effect size which can be used in a-priori power studies; we fail to see how such an accomplishment could be done with logistic regression. Finally, c) the ANOPA lead to simple expressions for standard error and confidence intervals for easily plotting the results, something impossible to achieve with logistic regression which is focused on coefficients, not on scores.

With all the logistic regression shortcomings reviewed above, and all the advantages of ANOPA, it is difficult to maintain that logistic regression is a superior technique to ANOPA.

References

Laurencelle, L. (2021b). Etude comparative de trois fonctions de transformation d'une proportion (Anscombe, 1948 – Freeman-Tukey, 1950 – Chanter 1975). *The Quantitative Methods for Psychology*, *17*, 166–194. doi: 10.20982/tqmp.17.3.p166

Wilks, S. S.(1938) The Large-Sample Distribution of the Likelihood Ratio for Testing Composite Hypotheses. *Annals of Mathematical Statististics*, *9* (1), 60--62. https://doi.org/10.1214/aoms/1177732360

Tables and Figures

Table B1: Best-fitting coefficients of a logistic regression on the data of the Second Illustration.

Coefficients:	β	Std. Error	z value	p value
Reference (SES=0 & Mod = 0)	1.6784	0.2911	5.765	< 0.0001
MoD_1 (from early to late)	0.6729	0.4707	1.430	0.1528
SES_1 (from low to middle)	-0.2593	0.4093	-0.634	0.5264
SES_2 (from low to hight)	-0.4745	0.4394	-1.080	0.2803
$\text{MoD}_1 \times \text{SES}_1$	-1.1365	0.6112	-1.859	0.0630.
$\text{MoD}_1 \times \text{SES}_2$	-1.1838	0.6335	-1.869	0.0617.

Note: MoD_1 = Moment of diagnostic Late.

Table B2. Small modification to the Second illustration to have more extreme results that can be detected by both the logistic regression and the ANOPA. The only changes are in the Middle-Late and High-Late conditions.

	Moment of diagnostic	
SES	Early	Late
Low	75 (89)	84 (92)
Middle	62 (77)	42 (72)
High	40 (52)	32 (63)

Table B3: Top: results of the logistic regression analyses on the data of Table B2 when (left) the level-0 condition is SES = low and (right) the level-0 condition is SES = middle; Middle: results of the ANOPA analyses on the same two coding. There are no differences in the ANOPA results. Bottom: Simple effect of the data of Table B2.

	SES Low is level 0				SES Middle is level 0			
Coefficients	β	Std. Error	z value	p value	β	Std. Error	z value	p value
Reference	1.6784	0.2911	5.765	<.0001**	1.41908	0.28774	4.932	<.0001**
MoD_1	0.6729	0.4707	1.430	.1528	−1.08261	0.37408	−2.894	.0038**
SES_1	−0.2593	0.4093	−0.634	.5264	0.25935	0.40934	0.634	.5264
SES_2	−0.4745	0.4394	−1.080	.2803	−0.21511	0.43718	−0.492	.6227
$\text{MoD}_1 \times \text{SES}_1$	−1.7556	0.6013	−2.920	.0035**	1.75556	0.60127	2.920	.0035**
$\text{MoD}_1 \times \text{SES}_2$	−1.8452	0.6272	−2.942	.0033**	−0.08961	0.55837	−0.160	.8725

	SES Low is level 0					SES Middle is level 0				
	SS	df	MS	F	p-value	SS	df	MS	F	p-value
Between group	0.1609	5				0.1609	5			
Moment (P)	0.0276	1	0.0276	7.9420	.0048**	0.0276	1	0.0276	7.9402	.0048**
SES (Q)	0.0890	2	0.0445	12.8001	<.0001**	0.0890	2	0.0445	12.8001	<.0001**
P × Q	0.0442	2	0.0221	6.3594	.0017**	0.0442	2	0.0221	6.3594	.0017**
Error	–	∞	0.0035			–	∞	0.0035		

	SES Low is level 0				
	SS	df	MS	F	p-value
SES effect for Early	0.0044	2	0.0022	0.6338	.4259
SES effect for Late	0.1289	2	0.0644	18.5256	<.0001
Error	–	∞	0.0035		

Note: Using William's correction for factor P, $c_P = 1.0011$, we obtain $p = .0049$, for factor Q, $c_Q = 1.0015$, we obtain $p < .0001$, and for factor P × Q, $c_{\text{P}\times\text{Q}} = 1.0066$, we obtain $p = 0.0018$.
MoD_1 = Moment of diagnostic Late; **: $p < .01$.
